# Antibacterial Crosslinker for Ternary PCL-Reinforced Hydrogels Based on Chitosan, Polyvinyl Alcohol, and Gelatin for Tissue Engineering

**DOI:** 10.3390/polym17111520

**Published:** 2025-05-29

**Authors:** Karina Del Angel-Sánchez, Ana Victoria Treviño-Pacheco, Imperio Anel Perales-Martínez, Oscar Martínez-Romero, Daniel Olvera-Trejo, Alex Elías-Zúñiga

**Affiliations:** Tecnologico de Monterrey, Institute of Advanced Materials for Sustainable Manufacturing, Ave. Eugenio Garza Sada 2501 Sur, Col. Tecnológico, Monterrey N.L., México City 64700, Mexico; kdelangel@tec.mx (K.D.A.-S.); a01137878@itesm.mx (A.V.T.-P.); anel.perales@tec.mx (I.A.P.-M.); daniel.olvera.trejo@tec.mx (D.O.-T.)

**Keywords:** hydrogels, forcespinning polycaprolactone fibers, gentamicin sulfate, articular cartilage replacement, non-Gaussian constitutive material model

## Abstract

Current hydrogels used for cartilage tissue engineering often lack the mechanical strength and structural integrity required to mimic native human cartilage. This study addresses this limitation by developing reinforced hydrogels based on a ternary polymer blend of poly(vinyl) alcohol (PVA), gelatin (GL), and chitosan (CH), with gentamicin sulfate (GS) as an antimicrobial agent and a crosslinker. The hydrogels were produced using two crosslinking methods, the freeze/thaw and heated cycles, and reinforced with forcespun polycaprolactone (PCL) nanofiber to improve mechanical performance. Chemical characterization revealed that GS forms weak hydrogen bonds with the ternary polymers, leading to esterification with PVA, and covalent bonds are formed as the result of the free amino group (-NH_2_) of chitosan that reacts with the carboxylic acid group (-COOH) of gelatin. SEM images help us to see how the hydrogels are reinforced with polycaprolactone (PCL) fibers produced via force spinning technology, while mechanical properties were evaluated via uniaxial tensile and compressive tests. Water retention measurements were performed to examine the crosslinking process’s influence on the hydrogel’s water retention, while the hydrogel surface roughness was obtained via confocal microscopy images. A constitutive model based on non-Gaussian strain energy density was introduced to predict experimental mechanical behavior data of the hydrogel, considering a non-monotonous softening function. Loading and unloading tests demonstrated that GS enhanced crosslinking without compromising water retention or biocompatibility because of the reaction between the free amino group of CH and the carboxylic group of gelatin. The PCL-reinforced PVA/GL/CH hydrogel shows strong potential for cartilage repair and tissue engineering applications.

## 1. Introduction

Tissue engineering is among the most promising technologies for meeting major clinical demands for bone and cartilage regeneration with sustainable properties [1]. The biomechanical content of articular cartilage is principally 80% water and approximately 20% organic matter, containing chondrocytes and extracellular matrix, major compounds of proteoglycans, and collagen of all types (I, II, III, and IV) [2,3,4]. Articular cartilage is an isolated organ with no lymphatic channels, blood vessels, or neurologic innervation, and its cell density is below that of other tissues. Therefore, it cannot fully repair structural damage from injury or disease [5]. The histological structure of cartilage allows it to disseminate shear, tensile, and compressive forces [6]. Cartilage defects, such as those seen in osteoarthritis, are a common cause of disability worldwide, not only affecting most of the population over 60 years, but also the lesions frequently occur in middle-aged and elderly patients, increasing the incidence of knee damage in adults, overweight people, and athletes with sports injuries is notable. Between 2012 and 2019, the American Joint Replacement Registry (AJRR) reported that around 2 million hip and knee arthroplasty procedures were performed in the United States, reflecting a 24.4% increase compared with previous years [7]. In 2020, approximately 595 million people were living with osteoarthritis, and it is projected that by 2050, this number will rise to 983 million individuals affected by osteoarthritis in the knee, hand, and hip, driving the growth of the regenerative medicine market [8].

Autografts or allografts are used in most bone implantations. Tissue regeneration can be successfully achieved using biomimetic materials that create an appropriate microenvironment for cell recruitment, adhesion, proliferation, and differentiation of cells [9,10]. However, allografting is limited by potential infection and a high non-union rate. Therefore, numerous strategies are currently being developed to avoid the use of bone grafts. Temporary scaffolds are necessary to regenerate bone and facilitate bone healing. Hydrogels form the soft skeleton of bone tissue engineering, providing hydrophilicity and flexibility [11]. Hydrogels are crosslinked networks of hydrophilic polymers that are insoluble in water, where the polymers are swollen but not solvated and contain either ionic or covalent crosslinks [12], chain entanglements, crystallites, hydrogen-bonded structures, and van der Waals forces acting as physical crosslinks in these systems [13].

Articular cartilage is a smooth white tissue covering the ends of two bones that form joints. It also serves as lubricating and energy-absorbing tissue that resists impact damage. Trauma, inflammatory causes, or aging can damage articular cartilage. These damages cause degradation of the extracellular cartilage matrix (ECM), decrease cellularity, and finally lead to osteoarthritis (OA) [14,15]. There are two types of cartilage imperfections, osteochondral and chondral defects, which may differ in their immune responses during allogeneic transplantation [16]. The immune response to chondrocytes can be triggered by contact with the bone marrow. Although the effect of immune reactions on clinical outcomes in osteochondral defects has not yet been determined, various strategies have been developed to regenerate functional cartilaginous tissues, such as abrasive chondroplasty, microfracture, and spongialisation. In addition, tissue transplantation with chondrogenic properties has also been proposed [17]. However, despite advances in orthopedic surgery, treatment for cartilage damage remains challenging. The several limitations associated with calcification and graft instability led to the development of porous scaffolds and tissue engineering. Natural and synthetic polymers are the primary resources for fabricating porous scaffolds for controlled drug release and surgical applications [18,19,20]. Particularly, polymers of natural origin form hydrogels that mimic the extracellular matrix (ECM), promote tissue growth and can regulate division, adhesion, differentiation, and migration of cells more favorably than synthetic polymers [21]. Lutzweiler et al. [22] claim that porous scaffolds are solid, three-dimensional structures with a porous architecture that allows infiltration, attachment, and proliferation of cells and tissue regeneration in a short time. Materials from natural origin, such as elastin [23], chitosan [24], cellulose [25], gelatin [26], hyaluronic acid [27], fibrinogen [28], collagen [28], sodium alginate (SA) [29], and starch [30] have been used for producing synthetic porous scaffolds. On the contrary, synthetic polymers such as poly(glycolic acid) (PGA) [31], PDMS25 hydroxyl silicone oil 25 cast, PDMS1000 hydroxyl silicone oil 1000 cast [32], polyvinyl alcohol (PVA) [33,34] polyethylene glycol diacrylate (PEGDA) [35], poly(lactic-co-glycolic) acid (PLGA) [36], and polycaprolactone (PCL) [37] have been the most used polymers for the fabrication of porous scaffolds. Among these materials, SA and starch are functional components used as scaffolds in tissue regeneration due to their biocompatibility, biodegradability, and tunable properties. However, their biomedical applications are limited by a few factors. One major issue is their low mechanical strength, which makes them unsuitable for applications requiring high structural integrity. Additionally, starch has limited bioactivity, affecting its ability to promote cell adhesion and differentiation, which is crucial for tissue engineering. Furthermore, rapid degradation of these materials can compromise the scaffold’s structural support before tissue regeneration is complete. The biomaterial used as a scaffold for tissue regeneration must be non-toxic, non-inflammatory, and biodegradable, able to provide mechanical structure and support, and biological conditions for cell growth, principally being accepted by the Food and Drug Administration (FDA). Material chemistry, molecular weight, solubility, shape and structure, hydrophilicity/hydrophobicity, lubricity, surface energy, water absorption, degradation, and erosion mechanisms are the key factors in selecting biomaterials for tissue engineering [38]. In addition, biodegradability, biocompatibility, plasticity, and adhesiveness of polymer-based hydrogels are extensively used in tissue engineering [39,40].

Gelatin is a water-soluble natural polymer obtained from various animal by-products that easily forms films and matrix hydrogels and is a partially degraded collagen product. Its fast biodegradation rate and retention of specific collagen information signals make it attractive for biomedical applications [41,42,43]. The chitosan molecule, a copolymer of D-glucosamine, is a material resembling cellulose in its solubility and low chemical reactivity. This polysaccharide is obtained by the alkaline deacetylation of chitin, a naturally abundant mucopolysaccharide, and the supporting material of crustaceans and insects [44]. It has excellent biocompatibility and biodegradability and forms a hydrophilic surface that enables easy cell adhesion, proliferation, and differentiation [45]. By contrast, PVA is a water-soluble synthetic polymer composed of vinyl monomer units. Due to its importance, the production of PVA has been heavily used to produce polymeric materials [46].

Hydrogels have been utilized for drug delivery, to produce scaffolds for tissue engineering, to replace native articular cartilage, and for wound dressings, to name a few [47,48,49,50,51,52,53]. They are also used as bone cement in orthopedic surgeries for the fixation of artificial implants. Hydrogels are water-swollen three-dimensional polymer networks composed of hydrophilic polymers crosslinked either through covalent bonds or held together via physical intramolecular and intermolecular attractions. They provide remarkable options for delivering cells with clinical applications [54], are superabsorbent materials that retain large amounts of water (>50%), and emulsification is the most used technique for fabricating hydrogel nano- and microparticles [55]. They are favorable as they offer the advantage of being injectable or preformed, and gelation can be induced through high or low temperatures, low pH, high ionic strength, overtaxing, sonication, freeze gelation, or electro-gelation [56,57]. Moreover, hydrogels play an essential role in cartilage replacement, but some challenges need to be addressed, such as attaining their desired mechanical response, stability, and self-healing properties. Recent progress in hydrogel developments for cartilage replacements is broadly discussed in [58,59].

On the other hand, the chemical crosslinking technique is a common method to prepare the hydrogels through covalent bonding [60,61]. However, it causes component leaching from the hydrogels. Therefore, a physical method of gelation and solidification of polymers has been developed to avoid component leaking from the traditional chemical crosslinking methods [62,63,64,65]. A physical crosslinking method provides a stable hydrogel through the crystalline regions of the polymers/materials. This technique promotes non-toxic and improved mechanical properties of hydrogels, making them suitable for biomedical applications. Curley et al. [66] suggest that hydrogels should ideally contain strong crosslinks and not easily revert from this state. Recent advancements in hydrogel formulations indicate that chemical or physical crosslinking can improve the mechanical properties of hydrogels and that the properties depend on the crosslinking agent. See [12,67,68,69] and references cited therein. Similarly, a dual network consisting of both physical and chemical crosslinks in ternary hydrogels has demonstrated excellent mechanical properties, high stretchability, and formability. This improvement is attributed to the synergistic effects between the different components of the hydrogel [70].

Recent research has emphasized the development of ternary hydrogel-based systems because of the benefits such as enhanced mechanical properties, synergistic functionalities, improved drug loading and release control, tunable physicochemical properties, improved stability, biocompatibility, and biodegradability. The reported ternary blends include combinations such as chitosan/guar gum/CMC, CMC/chitosan/PVA, SA/CMC/bioactive glass, CMC/carboxymethyl chitosan/carboxymethyl β-cyclodextrin, and gelatin/oxidized sodium alginate/PVA. These systems are being extensively studied for their chemical and mechanical properties, with promising applications in drug delivery, wound healing, tissue engineering, and biomedical scaffolding [71,72,73,74,75]. Particularly, research on ternary hydrogels based on PVA, gelatin, and chitosan is increasing because of their biocompatibility, mechanical stability, and functional versatility, making them highly suitable for biomedical applications [76,77,78,79,80,81].

On the other hand, GS is an antibiotic used in hydrogels because of its broad-spectrum aminoglycoside antibiotic that leaves the bacterium unable to synthesize proteins vital for its growth [82]. Moreover, GS provides antibacterial inhibition when the hydrogel is hydrated, is highly soluble in water, possesses good thermal stability, is not toxic for cell culture, and reduces bacterial contamination [83]. Furthermore, this antibiotic is used to treat infections caused by bacteria, such as meningitis, and infections of the blood, abdomen, lungs, skin, bones, articular joints, and urinary tract [84,85]. Therefore, GS as a component added to the hydrogels to provide antimicrobial properties and drug release has been widely studied [86,87,88,89,90]; however, there are fewer investigations about its role as a crosslinker [91].

This study discusses, for the first time, the development of hydrogels for cartilage tissue engineering using a ternary polymer blend (PVA, gelatin, chitosan) and gentamicin sulfate (GS) as an antimicrobial crosslinker. In addition to GS, two crosslinking methods, the freeze/thaw and heated cycles, were used to solidify the gel and form a hydrogel. The produced hydrogels were reinforced with forcespun polycaprolactone (PCL) fibers to improve their mechanical strength to sustain loads similar to those of the human native articular cartilage. The development of reinforced hydrogels focuses on mimicking the structural properties (compressive stress versus stretch) of human native cartilage, elucidating their physical behavior, and providing an alternative for use in cartilage repair and regeneration engineering. Additionally, a non-Gaussian constitutive model was proposed to predict mechanical behavior.

## 2. Materials and Methods

### 2.1. Material

Gelatin from porcine skin powder-gel strength 300 g Bloom Type A, chitosan low molecular weight Deacetylated chitin Poly(D-glucosamine), polycaprolactone (PCL, average $M_{n}=$ 80,000 g/mol), and poly (vinyl alcohol) with a molecular weight in the range of 89,000–98,000 were purchased from Sigma-Aldrich (St. Louis, MO, USA). Acetic acid reagent plus ≥ 99%, gentamicin sulfate injectable solution 160 mg/2 mL, and sodium hydroxide ≥ 97% were purchased from AMSA Lab (México City, México) and Jalmek (Monterrey, México), respectively.

### 2.2. Fiber Meshes Preparation

**Figure 1:**
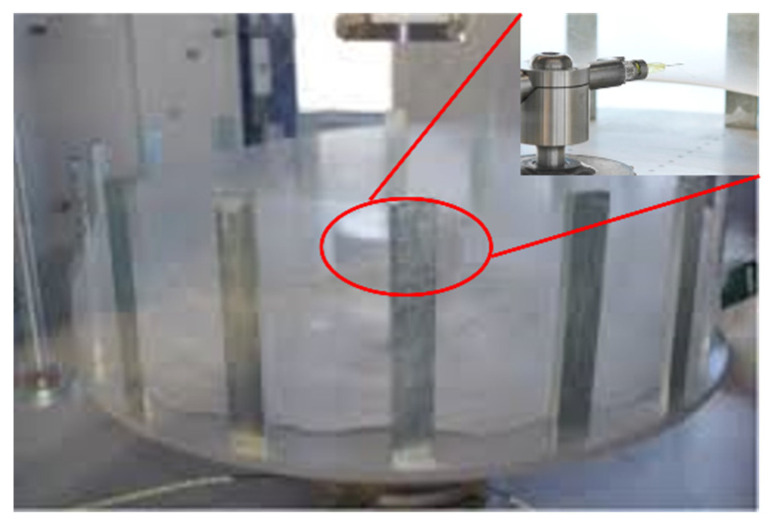
Spun PCL fibers by the Forcespinning technique. The inset figure marked with a red circle shows the spinneret that contains the solution for fabricating the PCL fibers.

PCL microfibers were produced using Forcespinning technology. During the fiber manufacturing process, we used PCL 13% *w*/*v*, which was sonicated for 15 min in chloroform. For all the materials, approximately 2 mL of solution was injected into a cylindrical spinneret using a 3 mL syringe with an 18-gauge × ½ inch needle. Additionally, 30-gauge × ½ inch needles were inserted into each side of the spinneret Forcespinning equipment. The angular velocity at which the fibers were spun was 6000 rpm for 1 min. The PCL fibers were collected on a 1.6″ × 1.6″ aluminum square collector with a 1.2″ × 1.2″ concentric void. Figure 1 depicts the PCL fibers collected on the crown during the Forcespinning process.

### 2.3. Hydrogel Preparation

PVA 20% *w*/*v* was sonicated for 15 min with distilled water and then magnetically stirred for 2 h. GL 30% *w*/*v* was dissolved in acetic acid, and chitosan 2.5% *w*/*v* was mixed with 2% acetic acid. These three materials were magnetically stirred together for 4 h without heating in a 10:2:1 ratio of (PVA:GL:CH). Then, the mixture was deposited in Petri dishes, and the fiber meshes created by forcespinning were immersed in this solution before the gelation process to create a sandwich-type hydrogel (STH). The collected fibers were arranged in Petri dish caps, maintaining the meshes parallel to the surface of the caps. Approximately eight grams of hydrogel solution were added to the Petri dish caps, fully embedding the meshes within the hydrogel.

Two different crosslinking methods were used; in the first method, the STH polymeric solution was kept in an oven at 50 °C overnight. For the second method, the sample was kept at –4 °C for 24 h, followed by one hour at room temperature, repeated for six cycles. Samples were labeled as LT (low-temperature) for the freeze/thaw cycles, HT (high-temperature) for the overnight heated hydrogels, and C and AB for the control samples without and with GS, respectively. When GS was used as a crosslinking agent, 1 mL (80 mg) of this compound was added to the PVA:GL:CH solution. The subsequent steps were conducted in the same way as for the other samples. The antibiotic is expected to be released into the body during the degradation process. The recommended dose of gentamicin sulfate for adults is 1–1.7 mg/kg every 8 h [92,93]. After the material was dried or frozen, 0.2 M NaOH solution was used to neutralize the hydrogels for 15 min. Finally, they were washed with distilled water until they reached a neutral pH.

### 2.4. Characterizations of the Hydrogels

#### 2.4.1. Structural Properties by XRD

The X-ray diffractometer PANalytical (X’Pert Pro PW1800, Almelo, The Netherlands) was used for the crystallographic analysis. The angle interval in 2ϴ used was from 10–70° with a step size of 0.10°/s by using a Cu Kα radiation (λ = 1.54 Å) with one nickel filter.

#### 2.4.2. Chemical Analysis by FTIR

Perkin Elmer Frontier Spectrometer (Waltham, MA, USA) was used to perform the FTIR measurements to identify the presence of specific chemical groups in the hydrogels. The samples of hydrogels of 4 mm thickness were analyzed using Attenuated Total Reflection (ATR) mode with a diamond point. The spectra were collected with wavenumbers ranging from 4000 to 400 cm^−1^ during 16 scans, with a resolution of 4 cm^−1^.

#### 2.4.3. Thermal Stability by TGA

Thermal stability was analyzed by thermogravimetric analysis (TGA) using Pyris 1 Equipment from Perkin Elmer (Connecticut, USA). The samples were heated from 50 to 500 °C under a nitrogen atmosphere of 20 mL/min at a heating rate of 10 °C/min and atmospheric pressure.

#### 2.4.4. Morphological Analysis by SEM

The morphology of the hydrogels was assessed by SEM using EVO MA 25 ZEISS (Oberkochen, Germany). The SEM images were taken at three different zones for each sample with an accelerating voltage of 5–10 kV in a high vacuum. Before capturing the images, the hydrogels were coated with a thin layer of gold to minimize electrostatic charge.

#### 2.4.5. Surface Roughness

The samples’ roughness was determined using a confocal microscope Axio CSM 700 (Jena, Germany). Roughness (R_a_) values were computed in 5 different sections of the samples’ surface, and the average was calculated.

#### 2.4.6. Mechanical Properties

Tensile tests and unconfined compressive tests were conducted using the INSTRON 3365 universal testing machine (Norwood, MA, USA), which has a load cell capacity of 5 N and 5 kN. The tests were performed at room temperature after the samples were hydrated for one hour with 20 mL of distilled water, following ASTM standards F2150. For the tensile testing, the samples were cut into strips, and the tests were conducted at a speed of 1.5 mm/min. For the compressive testing, the samples were shaped into rectangular prisms, and the tests were performed at a speed of 1 mm/min under a limited strain of 60%. Five tests were conducted for each sample, and the average results were reported.

#### 2.4.7. Contact Angle Measurements

The contact angle technique was performed employing a Contact Angle Data Physics equipment model OCA15EC (Filderstadt, Germany). Distilled water was deposited over the hydrogel surface to determine its hydrophilic behavior.

#### 2.4.8. Water Retention Analysis

The percentage of water retention was calculated using an analytical balance based on the weight difference at the beginning and the end of the 29 h test, with water as the liquid used for measurement. The percentage of water retention was calculated using Equation (1) [94], where $W_{h}$ represents the humid weight and $W_{d}$ represents the dry weight.

(1) $$\mathrm{Water}\;\mathrm{Retained}=\frac{W_{h\;}-W_{d}}{W_{d}}\times 100$$

#### 2.4.9. Cell Growth Tests

Hydrogels were washed three times with phosphate buffer saline (PBS, pH 7.3). The sterilization process was conducted using a short-wave ultraviolet light for three hours. According to the protocol, the incubation was made using Dubelcco’s Modified Eagle’s Medium (DMEM, MA, USA) for 24 h. Hydrogels were incubated in microplates with 24 holes, and 20,000 stem cells derived from adipose tissue were seeded per hole. The microplates were incubated at 37 °C with 5% CO_2_ gas inside a humid chamber, changing the medium every third day for three weeks. The qualitative cell growth was observed through an inverted microscope (Optika, Ponteranica, Italy) for each medium change.

## 3. Results and Discussions

### 3.1. Hypothetical Reaction Mechanism of PVA/GL/CH Hydrogel with Gentamicin Sulfate as Crosslinker

**Figure 2:**
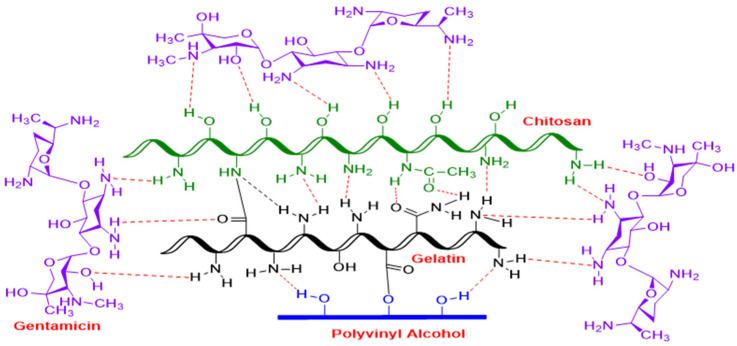
Hypothetical mechanism reaction of PVA/GL/CH Hydrogel crosslinked with gentamicin sulfate. Dashed lines indicate the chemical interactions of functional groups between the components.

During the hydrogel formation based on PVA/GL/CH, some reactions are responsible for forming covalent bonds. The free amino group (-NH_2_) of CH reacts with the carboxylic acid group (-COOH) of gelatin to form a covalent amide bond (NCO). As shown in Figure 2, weak hydrogen bonds can occur between the free amino groups (-NH_2_) of chitosan and gelatin. GL and PVA can also form a covalent bond when the reaction between the carboxyl acid of GL and the free alcohol (-OH) of PVA forms ester bonds (-COO-). In addition, weak hydrogen bonds can be formed between the free amino groups of gelatin and the alcohol functional groups of PVA. After adding an antibacterial agent (gentamicin sulfate) to the ternary PVA/GL/CH mixture, GS can interact with all the components [43,95,96,97]. It can form weak hydrogen bonds through its unprotected and protected amino functional groups. Crosslinked protein-based hydrogels with hydrogels based on polymeric matrix have been studied in the past. Wang et al. [98] reported multiple hydrogen bonds, dynamic covalent bonds, and electrostatic interaction between CS, PVA, and *p*-carboxyphenylboronic acid as a result of physical and chemical crosslinking. Utterström et al. [99] found that the pH and environmental temperature can affect the hydrogel in different ways according to the placement of the coiled-coil segments’ amino acid structure and the hydrophilic protein domains, which can be manipulated to improve cell interactions [43].

### 3.2. Crystallographic Analysis by X-Ray-Diffraction (XRD)

**Figure 3:**
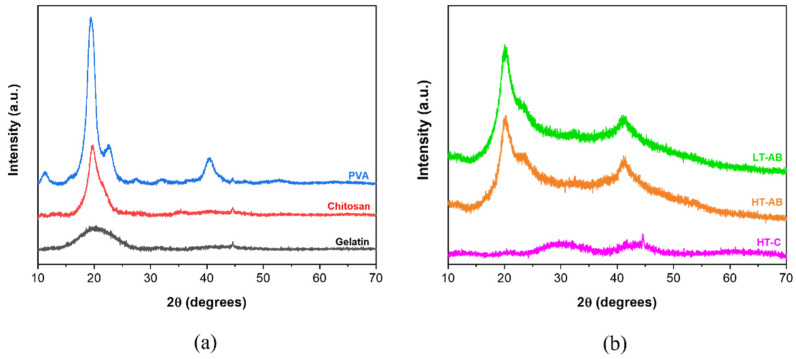
(**a**) XRD patterns of precursors of hydrogel: gelatin, chitosan polyvinyl alcohol, and (**b**) hydrogels crosslinked with gentamicin at low temperature (LT-AB), high temperature (HT-AB), and with no gentamicin (HT-C).

Figure 3 shows the typical X-ray diffractograms of ternary compound PVA/GL/CH hydrogels and their precursors. The pure precursors were analyzed in powder, as shown in Figure 3a. The amorphous nature of gelatin is evidenced by a broad peak at 19.3–20.0° in 2θ [100]. A high-intensity peak at 20.0° in 2θ is associated with chitosan (red plot) [100]. PVA exhibits peaks located at 11.0°, 19,4°, 22.6° and 40.3° [101,102]. The highest intensity corresponds to *hkl* (101) plane, indicating the semi-crystalline nature of PVA [103,104]. The diffractogram pattern of the HT-C sample only depicts a broad shoulder from 25.0° to 33.0° in 2θ and a small peak in 2θ = 44.5° that matches the precursors (gelatin, chitosan, and PVA). This can be evidence that a weak crosslink occurs in the absence of gentamicin compared with hydrogels with GS. The diffractograms of the samples crosslinked at low and high temperatures showed nearly identical peak patterns and positions. In the diffractogram of the HT-AB (high temperature with GS), three characteristic peaks were observed at 20.2°, 23.3°, and 41.2° in 2θ. These peaks are related to the crystalline phase of hydrogels. Among them, one high-intensity peak was observed at 20.02°, which overlapped with PVA and CH peaks, and another low-intensity peak was observed at approximately 23.0°. The last peak was detected at 41.0° with medium intensity.

### 3.3. Fourier-Transform Infrared Spectroscopy Measurements

**Figure 4:**
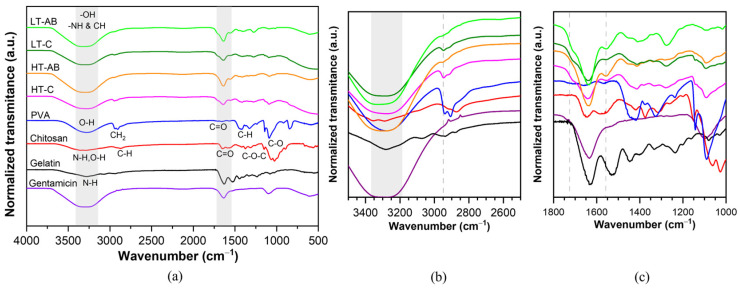
(**a**) FTIR spectra of the hydrogel precursors, including gelatin, chitosan, polyvinyl alcohol, and gentamicin sulfate. The spectra also show the hydrogels that were crosslinked at high temperature without gentamicin (HT-C), with gentamicin (HT-AB), and those crosslinked at low temperature without gentamicin (LT-C), and with gentamicin (LT-AB). (**b**) A zoomed-in view of the region from 3500 to 2500 cm^−1^ of (**a**). (**c**) A zoomed-in view of the region from 1800 to 1000 cm^−1^.

The main absorption bands of raw materials for obtaining hydrogels based on PVA/CH/GL were observed by FTIR analysis, as shown in Figure 4. For raw PVA, the bands are assigned as follows [105,106]: O-H stretching (3276 cm^−1^), CH_2_ asymmetric stretching (2945 cm^−1^), C–H symmetric stretching (2904 cm^−1^), CH_2_ bending vibration (1445 cm^−1^), C–H symmetric stretching (1324 cm^−1^), CH_2_ wagging or CH wagging (1230 cm^−1^), CH_2_ wagging (1140 cm^−1^), C–O–C stretching vibrations (1093 cm^−1^). The bands between 918 and 600 cm^−1^ were identified as CH_2_ rocking, C–C stretching, and OH wagging. The C=O stretching at 1657 cm^−1^ corresponds to acetate residual from partially hydrolyzed PVA [107]. The gelatin compound has a similar band to PVA [96], attributed to the CH_2_ bending vibration located at 1443 cm^−1^. The N-H stretching from amide I, II, and III appears at 1630, 1529, and 3284 cm^−1^. Signals of the C=O stretching from carboxylic acid can overlap with Amide I. Chitosan bands were assigned according to O-H, N-H stretching, and intramolecular hydrogen bonds at 3300 cm^−1^, C–H symmetric and asymmetric stretching at 2922 cm^−1^, N-H bending of amide II at 1572 cm^−1^. The C=O group at 1646 cm^−1^ is due to the presence of residual N-acetyl groups [108]. On the other hand, the PVA/CH/GL hydrogel samples exhibited broad peaks at 3325 cm^−1^, which were attributed to the overlapping signals from the hydroxyl groups of PVA and the hydroxyl/amine groups of chitosan (-NH of CH). Campaña et al. [109] reported similar findings regarding overlapping signals in the range of 3200 to 3500 cm^−1^, which were attributed to -OH and -NH groups. The chemical interaction of the free amino group (-NH_2_) of chitosan with the carboxylic acid group (-COOH) of gelatin forms covalent amide bonds (NCO), and they can be evidenced with a short band at 1554 cm^−1^ (see Figure 4c). The peaks observed at 1279 cm^−1^ and 1409 cm^−1^ were assigned to the CH_3_ symmetrical deformation mode resulting from the C-H vibration. Weak hydrogen bonds can be overlapped in the region of 3300–3450 cm^−1^ with the free amino groups (-NH_2_) of chitosan and gelatin when they react, and a short band around 1730 cm^−1^ can also be attributed to weak hydrogen bonds as a result of the chemical interaction between precursors (PVA, CH, and GL). Similar results about weak hydrogen and covalent bonds confirmed by FTIR for a ternary hydrogel based on PVA, CH, and GL were reported [81]. Regarding the chemical interaction of gentamicin sulfate, at 2950 cm^−1^, both HT-AB and LT-AB samples exhibit similar behavior, distinguishing them from the –C samples by a decrease in band intensity (see Figure 4b). The peak at approximately 600 cm^−1^, present in -AB samples, indicated the C-C bond stretching of CH. This can be evidence that a weak crosslink occurs in the absence of gentamicin compared with hydrogels with GS, as was observed by XRD. It is important to highlight that the behavior was basically the same for the four different samples, except for the signals at 2950, 1730, and 600 cm^−1^. Very similar results were observed by Lihong Fan et al. [110].

### 3.4. Thermal Stability Analysis

**Figure 5:**
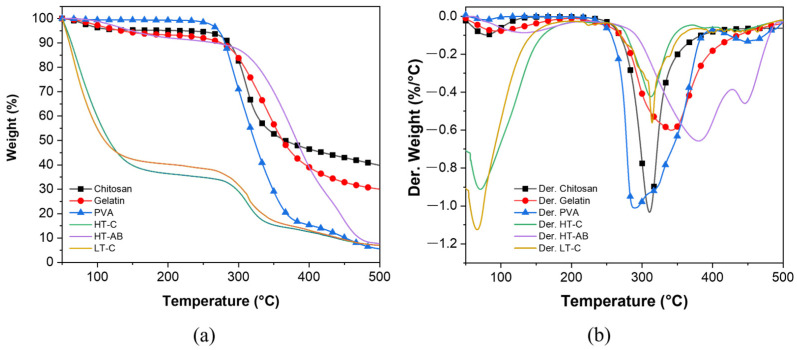
(**a**) Thermograms of chitosan, gelatin, and PVA in powder and the hydrogels crosslinked at high and low temperatures. (**b**) Derivative TGA results.

The thermal stability of hydrogels and the precursors in powder was analyzed through the change in weight according to temperature increment, and the results are depicted in Figure 5. Pure chitosan presents two main degradation steps at 78.3 °C and 310.5 °C; the first is attributed to the loss of water that polysaccharides contain because of their hydrophilicity character, and in the second stage occurs the scission of the ether linkage in the chitosan backbone [111]. Gelatin powder has a similar behavior to chitosan; it is decomposed in two steps: the first, in the range of 50 °C to 150 °C, is due to moisture weight loss, and the second, in the range of 250 °C to 400 °C, represents the disintegration of protein chains [112,113]. Table 1 summarizes data obtained in the TGA and DTGA plots; T_10%_ represents the temperature at which the samples lose 10% of their weight, T_p_ is the peak decomposition temperature, and R_p_ denotes the peak decomposition rate. Note that the PVA powder exhibits three peak decomposition temperatures; however, only the two highest intensities are listed in Table 1. Due to the elimination of humidity, the first step decomposition temperature is observed at 74 °C; the second primary loss step is assigned to the breakdown of the chains with -OH groups, and the third step observed between 400 and 500 °C is ascribed to polymer decomposition and carbonization [114]. The hydrogels crosslinked at high and low temperatures (HT-C and LT-C, respectively) show similar thermal behavior, as shown in Figure 5a. Both hydrogels have two peak decomposition temperatures: 70.6 °C and 312.7 °C for the HT-C sample and 66.2 °C and 314.5 °C for the LT-C sample. Similarly, T_10%_ values were observed at 63.1 °C and 60.1 °C. The hydrogel crosslinked with gentamicin (HT-AB) indicates higher thermal stability than those without GS (see Table 1). Although the T_10%_ is smaller (266.4 °C) than for the precursors, which is in the range of 275–286 °C, it can be due to the absorbed water in the hydrogel. However, T_p1_ was incremented around five times for the hydrogels crosslinked at high temperatures with gentamicin as the crosslinker (HT-AB). Similarly, the T_p2_ was higher than that of hydrogels crosslinked without GS. This thermal stability improvement can also be evidenced by following the peak decomposition rate; higher thermal stability results in a lower decomposition rate, and vice versa. This behavior can be due to the chemical crosslink induced by GS since, according to the reaction mechanism, it can interact with all the precursors (PVA, chitosan, and gelatin) and can form weak hydrogen bonds from its unprotected and protected amino functional groups, covering thus the precursor molecules and delaying its decomposition. It is important to note that since all TGA tests were conducted under the same conditions, the generated residue can be attributed to incomplete combustion caused by the inert atmosphere during the test, which results in a solid carbonaceous residue.

### 3.5. Morphology by Scanning Electron Microscopy

**Figure 6:**
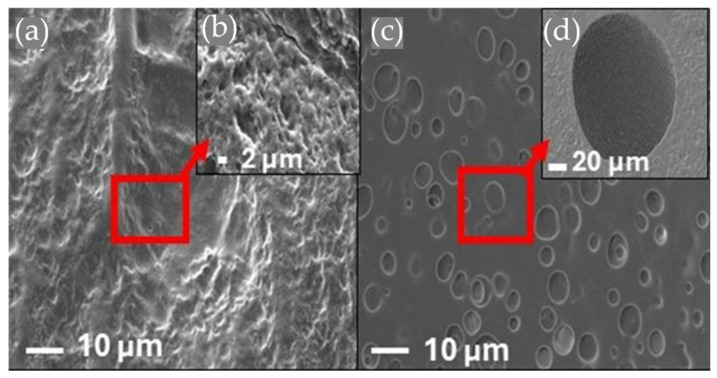
Morphological analysis of (**a**,**b**) LT-AB hydrogel and (**c**,**d**) HT-AB hydrogel.

The surface morphology of HT-AB and LT-AB samples analyzed using SEM imaging with magnifications of 2.5x and 5x are depicted in Figure 6. Each sample showed a different morphology. Figure 6a,b represent the morphology of the LT-AB sample; these show a pore-bearing surface, which could be due to the formation of physical bonds and the contraction of the mixture. The LT-AB sample chains from the polymeric network are contracted. The freeze/thaw cycles serve as an extra physical crosslink due to crystallite formation. The number and stability of these crystallites increased as the number of freezing/thawing cycles increased [115]. Figure 6c,d illustrate segregation at the surface of HT-AB hydrogel caused by the high temperature used in the drying process, and the surface is smoother than that of the hydrogel crosslinked at freezing/thawing cycles.

**Figure 7:**
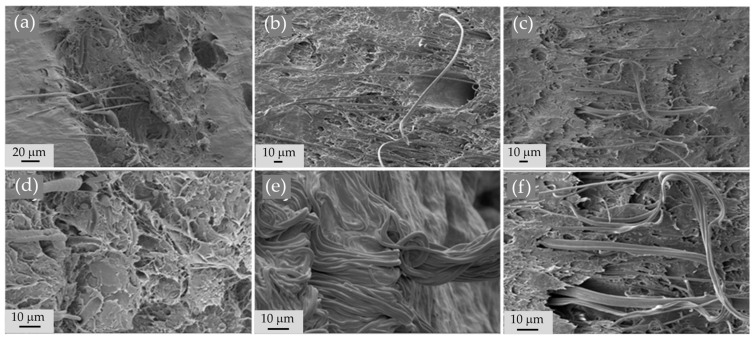
SEM images for hydrogels reinforced with PCL fibers with gentamicin to different volumes (**a**) 1 mL, (**b**) 1.5 mL, (**c**) 2 mL at 1000x magnification. (**d**) 1 mL, (**e**) 1.5 mL, (**f**) 2 mL at 2500x magnification.

Figure 7 illustrates SEM images of hydrogels reinforced with PCL fibers that have an average diameter of 1.3 µm. The volume of gentamicin used varied between 1 mL and 2 mL. Notably, the fiber structure is preserved within the hydrogel matrix, and the amount of gentamicin does not affect the reinforcement provided by the fibers. The morphology of the fibers remains consistent regardless of the gentamicin volume, and they interweave with the hydrogel structure. This characteristic is beneficial, as it enhances the hydrogels’ ability to support external loads. Small fiber diameter increases the surface area-to-volume ratio, which improves the interfacial bonding between the fibers and the hydrogel matrix [116].

### 3.6. Hydrogels’ Surface Roughness

**Figure 8:**
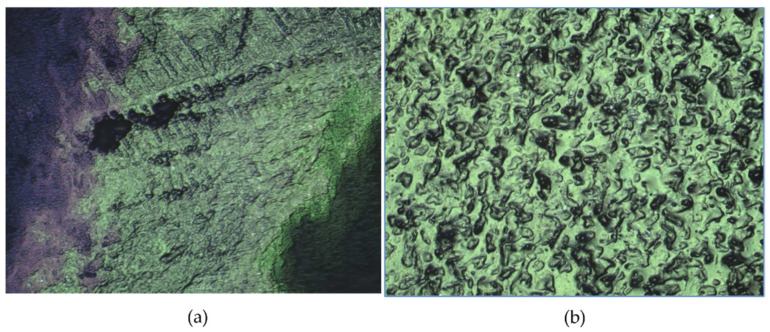
Surface morphology of hydrogel. (**a**) LT-AB hydrogel and (**b**) HT-AB hydrogel.

The roughness images of the hydrogels obtained by confocal microscopy are shown in Figure 8, while Table 2 provides roughness values and their comparison with other reported works. Material surface roughness directly influences cellular morphology, proliferation, and phenotype expression in vitro and in vivo tests [117,118]. Elshazy et al. [119] reported that hydrogel surfaces with roughness ranging from 27 to 250 μm exhibited good adhesion strength for Bovine Aortic Endothelial Cells (BAOEC). Notably, the samples with a roughness of 149 μm demonstrated a significant improvement in adhesion strength. In this study, the roughness measurements for the HT-AB and LT-AB samples were found to be between the ranges typical for articular cartilage and microroughness, specifically 2.5 μm and 2.7 μm, respectively, as shown in Table 2. This property is crucial for promoting adhesion and cell growth of mesenchymal stem cells. The rugosity correlates with the surface morphology and porosity observed by SEM; lower rugosity results in a smoother surface (HT samples), while higher rugosity leads to increased porosity (LT samples).

### 3.7. Mechanical Tests

**Figure 9:**
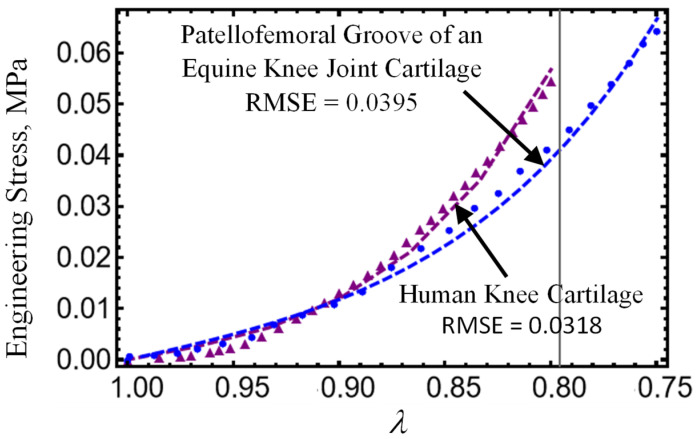
Stress versus stretch curves the patellofemoral groove of an equine [65] and the knee joint and human chondral cartilage knee layer [127]. Symbols (circle and triangle) indicate experimental data, and dashed lines indicate predicted results from the constitutive model given by Equation (7). Here, the material parameters found to fit data using Equation (7) are for equine cartilage: μ = 0.2 Mpa, N_8_ = 5.5, f = 0.16, A_1_ = −1.35 MPa, A_2_ = 2 MPa, and for human cartilage: μ = 0.2 MPa, N_8_ = 5.5, f = 0.195, A_1_ = −1.1 MPa, and A_2_ = 4.05 MPa.

First, the response to the loading of native cartilage needs to be explored, considering experimental data available in the literature. Nowadays, there are several references available in the literature that have investigated the mechanical response of native human cartilage coming from cadavers or human remaining tissue from surgical cartilage transplantations. See [122,123,124,125,126,127,128,129,130,131,132,133] and references cited therein. From experimental data reported in these references, the native cartilage Young’s modulus values are in the interval of 0.25 MPa to 3 MPa [122]. That could be influenced by the histologies of the donors, as discussed by Kos et al. [126,128], and by the articular cartilage biomechanics and the multi-scale nature of its mechanical behavior [122,134]. Figure 9 shows data of compressive stresses versus stretch curves collected from the knee joint and the human chondral cartilage knee layer [127] compared with data collected from the patellofemoral groove of an equine [65]. Notice the great similarity between the equine and human cartilages since the compressive stress versus strain data are similar within the same range [135].

**Figure 10:**
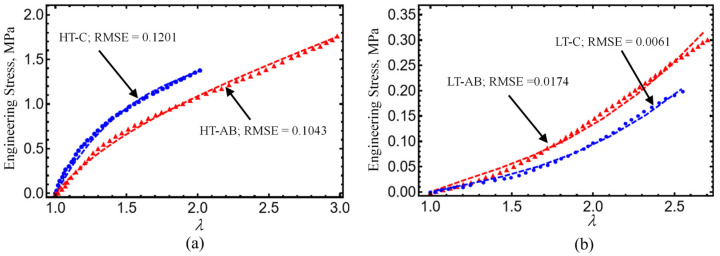
Uniaxial extension stress–stretch curves of hydrogels crosslinked at (**a**) high temperature (HT) and (**b**) low temperature (LT). Symbols are experimental data, while dashed lines are simulation results obtained from Equation (7). The material parameter values used to fit data with simulations results computed from Equation (7) were for (**a**) HT-C-AB and HT-C: μ = (0.25, 0.2) MPa, N_8_ = (2.5, 10.5), f = (0.075, 0.035), A_1_ = (14.5, 18.5) MPa, and A_2_ = (−4.5, −0.5) MPa, for (**b**) LT-AB and LT-C: μ = (0.195, 0.205) MPa, N_8_ = (60, 30.5), f = (0.0145, 0.019), A_1_ = (−15.5, −14) MPa, and A_2_ = (2.45, 1.5) MPa. Here, HT-AB and LT-AB are the hydrogel samples reinforced with GS.

Next, the response of the developed hydrogels is assessed by performing tensile and compressive tests to investigate how close their mechanical response is to that of human articular cartilage. First, tensile tests were conducted to analyze how gentamicin sulfate affects the crosslinking of hydrogels and compare the two gelation methods. The results are shown in Figure 10 and are summarized in Table 3. It is noteworthy that the tensile strength of the hydrogels crosslinked at elevated temperatures was significantly greater than that of those crosslinked at lower temperatures. This observation can be supported by the water retention capacity of crosslinked hydrogels which is discussed in Section 3.9. Since the water retention and crosslink density in a material are generally inverse and nonlinear, in this work, the hydrogels crosslinked at low temperatures presented higher water retention, which suggests that there are fewer crosslinks and more space between polymer chains for water penetration and retention, but low mechanical strength. Furthermore, a distinct variation in ultimate tensile strength was observed between the hydrogels crosslinked with gentamicin sulfate and those without for both gelation methods. Specifically, the hydrogels containing gentamicin sulfate exhibited enhanced strength and flexibility. In this respect, it appears that water retention is not directly related to the crosslinker used or the mechanical properties. Yurong Liu et al. worked with physically crosslinked PVA/GL hydrogels and reported an ultimate tensile strength of 0.07 MPa [64], lower than the ultimate tensile strength of LT-C hydrogel (0.203 MPa) and 0.303 MPa for LT-AB hydrogel. The percentage of improvement between LT-AB and LT-C samples can be computed using the expression:

(2) $$\mathrm{Percent}\;\mathrm{of}\;\mathrm{Improvement}=\frac{\sigma_{AB}-\sigma_{C}}{\sigma_{C}}\times 100$$

where $\sigma_{AB}$ is the ultimate strength in LT-AB samples, and $\sigma_{C}$ is the ultimate strength in LT-C samples. According to the results, hydrogels crosslinked with GS at high and low temperatures (HT and LT) exhibited an improvement of 27.3% and 49.2%, respectively, in their ultimate tensile strength values.

**Figure 11:**
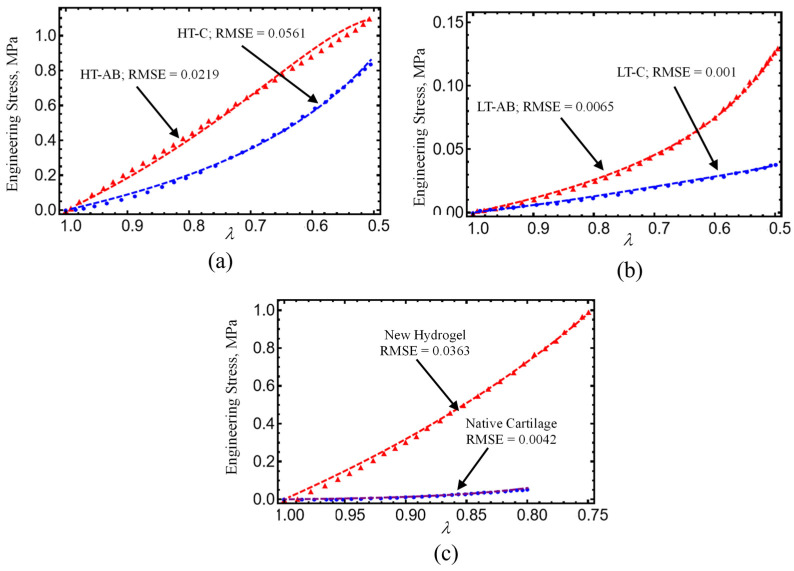
Compressive strength data of hydrogels crosslinked at (**a**) high temperature (HT), (**b**) low temperature (LT), and (**c**) native cartilage. Symbols are experimental data, while dashed lines are simulation results obtained from Equation (7). The material parameter values used to fit data with simulations results computed from Equation (7) were for HT-AB and HT-C: μ = 0.2 MPa, N_8_ = 3.5, f = (0.16, 0.09), A_1_ = (4.15, 1.25) MPa, A_2_ = (−3.5,−0.75) MPa, for LT-AB and LT-C: μ = 0.2 MPa, N_8_ = 3.5, f = 0.16, A_1_ = (−1.35,−1.5) MPa, and A_2_ = (−0.195, −0.3) MPa, for HD and the human native cartilage: μ = 0.2 MPa, N_8_ = (3.5, 5.5), f = (0.16, 0.195), A_1_ = (7.25, −1.1) MPa, and A_2_ = (0, 4.05) MPa.

Unconfined compressive tests were also performed to analyze the effect of gentamicin sulfate and the two different gelation methods employed. Figure 11 illustrates the compressive strength analysis performed on HT and LT samples. Similarly, as was observed in tensile stress, hydrogels crosslinked at high temperatures show a higher performance compared with LT samples without GS. Hydrogels crosslinked with GS exhibit compressive properties of 1.191 and 0.131 MPa for HT-AB and LT-AB samples, respectively. These values are higher than those reported in the literature for human native cartilage, as summarized in Table 3.

Equation (2) was used to calculate the improvement of compressive strength in the LT-AB samples. HT had 29.9%, and LT resulted in 244.7%. When the results were compared, in the tensile and compressive tests, HT samples showed better properties in all performed tests, but the gentamicin sulfate had a greater effect on LT samples, being the greatest effect on the compressive strength, resulting in an improvement of almost 250% comparing the LT-AB to the LT-C samples. HT-AB showed similar improvement in both tensile and compressive tests.

We introduce a material model to predict experimental data collected from compressive uniaxial loading and unloading tests of the developed hydrogels to determine their Young modulus value that will be compared with those of the native articular human cartilage values listed in the literature.

### 3.8. Contact Angle Tests

**Figure 12:**
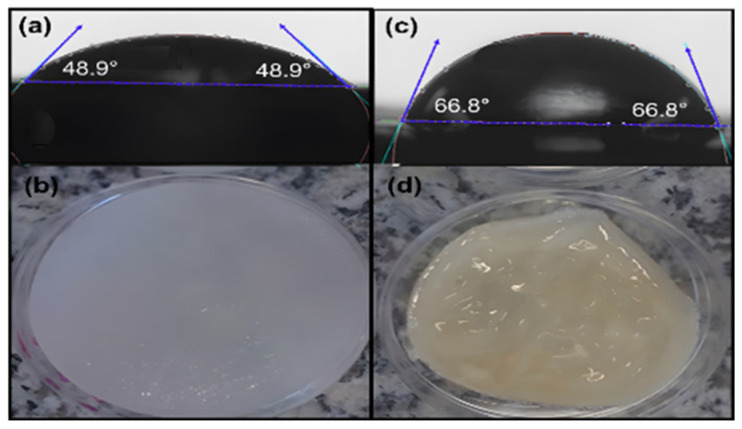
Contact angle images of (**a**) LT-hydrogel and (**c**) HT-hydrogel crosslinked with gentamicin sulfate. (**b**) and (**d**) images of hydrogels evaluated in (**a**) and (**c**), respectively.

We measured the moisture absorption capacity of the hydrogels by performing contact angle tests with water as the solvent. Figure 12 shows the results of hydrogels crosslinked with GS at low and high temperatures. Since a material with a contact angle < 90° is hydrophilic, the hydrogels developed at low and high temperatures showed this behavior. However, a remarkable difference between the physical crosslinking method was observed; the LT samples exhibited a lower contact angle (48.9°) than HT samples (66.8°). This result can be attributed to the hydrogel’s surface morphology and porosity observed by SEM, the LT-sample’s pore-bearing surface easily allows the absorption and water retention because of the exposed surface area. In contrast, the smoother surface of HT samples reduces the wettability.

### 3.9. Hydrogels Water Retention Tests

**Figure 13:**
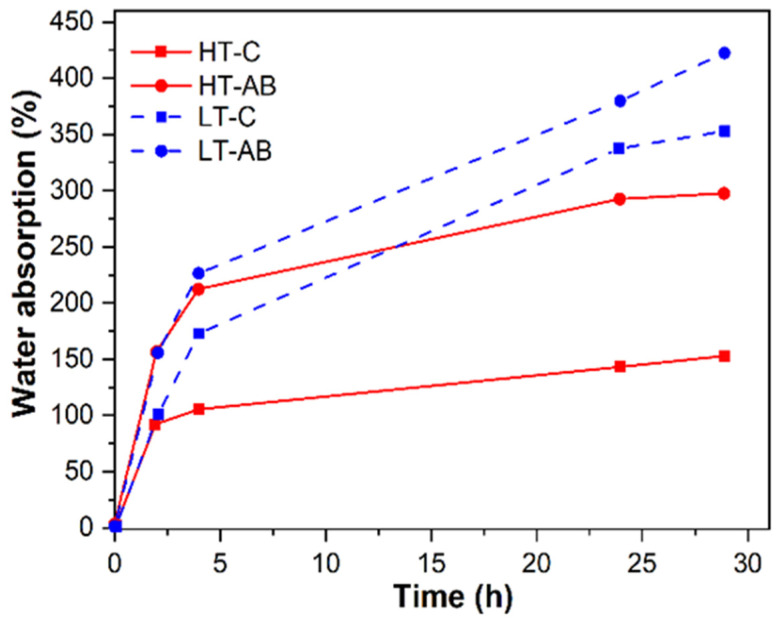
Water absorption evaluation of hydrogels crosslinked at low and high temperatures with and without gentamicin sulfate.

According to the potential applications of hydrogels, such as in articular cartilage, liquid retention is a crucial characteristic that enables proper functioning within the microenvironment. This factor was measured using water as liquid, and the test lasted 29 h. As shown in Figure 13, both HT-AB and LT-AB samples had similar water retention during the first 2.5 h; after this time, the LT-AB hydrogel had a greater water retention. Both -AB samples registered a higher water retention percentage than the HT-C and LT-C samples. It is important to mention that gentamicin sulfate in the LT-AB samples has a better effect on water retention than the HT-AB samples. Overall, LT-AB samples absorbed more water compared with the HT-AB samples. Water retention improvement was calculated using Equation (2). HT-AB and HT-C samples resulted in an improvement of 87% compared with 16.3% of the LT-AB and LT-C samples. Several factors contribute to water retention capacity, such as porosity, hydrogen bonding, and crosslink density. In general, all the samples showed a positive response to water retention behavior, which can be due to the weak hydrogen bonds of the ternary hydrogel-forming hydrogen bonds with water molecules. However, the lower response was for the hydrogel without gentamicin (HT-C), and this can be attributed to the fact that gentamicin favors the formation of hydrogen bonds, as was observed by FTIR analysis. In addition, according to the results determined by SEM, the porosity in LT-hydrogels is higher than that of HT-hydrogels, which can favor the absorption and retention of water. The improvement of water retention of hydrogels crosslinked with GS compared with those with no GS can be due to a higher amount of hydrogen bonds present as a result of interactions of gentamicin with the PVA, chitosan, and gelatin.

### 3.10. Cell Growth of Hydrogels

**Figure 14:**
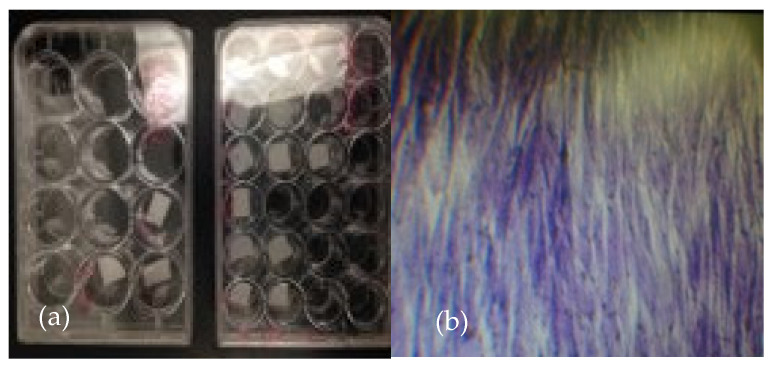
(**a**) LT-AB hydrogels incubated in microplates with 24 holes and 20,000 stem cells seeded per hole. (**b**) Cell growth of (**a**) after the incubation period.

Figure 14 illustrates the result of LT-AB samples as a scaffold material, a biocompatible and bioabsorbable extracellular matrix that possesses antimicrobial properties. This scaffold was successfully tested for its ability to promote the growth of stem cells and facilitate their differentiation into chondrocytes. As can be seen in Figure 14b, after the incubation period, cell growth was observed. Stem cells are unspecialized cells with a remarkable ability to differentiate into various specialized cell types, which combined with their capacity for self-renewal, makes stem cells a significant focus in the field of regenerative medicine [138]. To assess biocompatibility, key markers of chondrogenic and hypertrophic differentiation were evaluated. Among the many therapeutic possibilities, one of the most promising is the use of stem cells for regenerating cartilage tissue [139]. Articular cartilage has a limited ability to repair itself due to its avascular nature. Therefore, differentiating stem cells into chondrocytes—the cells responsible for cartilage formation—offers great potential for treating conditions such as osteoarthritis and cartilage injuries [15].

## 4. Constitutive Material Model

### 4.1. Material Model Formulation

To predict the material response of the hydrogels under uniaxial loading and unloading cycles, we adopt the material model introduced by Elías-Zúñiga et al. in [140]. This material model, based on the rule of mixtures and non-Gaussian strain energy density expressions, captures the behavior of biological materials by incorporating a non-monotonous softening function and the use of an isotropized representation form of the non-Gaussian eight-chain model that captures the material anisotropic effects. In this model, the Cauchy stress components are given as:

(3) $$T_{i}-T_{j}=\left\{\left(1-f\right)ℵ+\frac{2f}{3}\left(A_{1}+\frac{2A_{2}}{3}\left(I_{1}-3\right)\right)\right\}\left(\lambda_{i}^{2}-\lambda_{j}^{2}\right)$$

where i≠j=1, 2, 3, $\lambda_{i}$ are the principal stretches, $T_{i}$ are the Cauchy stress components, f represents the volumetric fraction, $A_{1}$ and $A_{2}$ are the isotropized material constants, $I_{1}$ is the principal invariant of the Cauchy-Green Deformation tensor given as:

(4) $$I_{1}=\lambda_{1}^{2}+\lambda_{2}^{2}+\lambda_{3}^{2}$$

ℵ is a material response function defined as:

(5) $$ℵ=\frac{\mu_{0}}{3\lambda_{r}}\left[\beta +\frac{1}{N_{8}}\left(\frac{1}{\lambda_{r}}-\frac{1}{\beta \left(1-\lambda_{r}^{2}-2\lambda_{r}/\beta \right)}\right)\right]$$

$N_{8}$ represents the material chain number of links [141], β is the inverse of the Langevin function $L\left(\beta \right)$ given as:

(6) $$\lambda_{r}=L\left(\beta \right)=\cot\beta -1/\beta =\sqrt{\left(\lambda_{1}^{2}+\lambda_{2}^{2}+\lambda_{3}^{2}\right)/3N_{8}}$$

and $\mu_{0}$ is the material shear modulus.

For incompressible materials under uniaxial tension and compression, we have that $\lambda_{1}=\lambda$, $\lambda_{2}=\lambda_{3}=1/\sqrt{\lambda }$, and $T_{2}=T_{3}=0$ For tensile loading λ≥1 and $T_{1}>0$, while for compressive loading 0<λ≤1 and $T_{1}<0$. From Equation (3), the Cauchy stress is given as:

(7) $$T_{1}=\left\{\left(1-f\right)ℵ+\frac{2f}{3}\left(A_{1}+\frac{2A_{2}}{3}\left(I_{1}-3\right)\right)\right\}\left(\lambda^{2}-1/\lambda \right)$$

while the uniaxial stress-softened constitutive material model follows the expression [142,143,144]:

(8) $$\tau_{1}=\left[\left\{\left(1-f\right)ℵ+\frac{2f}{3}\left(A_{1}+\frac{2A_{2}}{3}\left(I_{1}-3\right)\right)\right\}\left(\lambda^{2}-1/\lambda \right)+\frac{n}{d}\left[\lambda^{n-1}\right]\left(\lambda^{n}-\Delta^{n}\right)-\lambda^{-\left(1+n/2\right)}\left(\lambda^{\left(-n/2\right)}-\Delta^{\left(-n/2\right)})\right)\right]e^{-b\sqrt{(M-m)(m/M)}}$$

where n is a constant, whose value is fixed as ½, b is a dimensionless softening constant, d is a residual strain constant, Δ is the maximum stretch value on the virgin material curve for which unloading of the virgin material begins, *m* is the current strain magnitude value defined as [78,80,81]:

(9) $$m=\sqrt{\lambda_{1}^{4}+\lambda_{2}^{4}+\lambda_{3}^{4}}\equiv \sqrt{\lambda^{4}+2/\lambda^{2}}$$

and

(10) $$M\equiv \sqrt{\Delta^{4}+2/\Delta^{2}}$$

The uniaxial engineering stress tensor is found using the relation $\sigma =TF^{-1}$ where **F** is the deformation gradient tensor.

### 4.2. Comparison with Experimental Data

**Figure 15:**
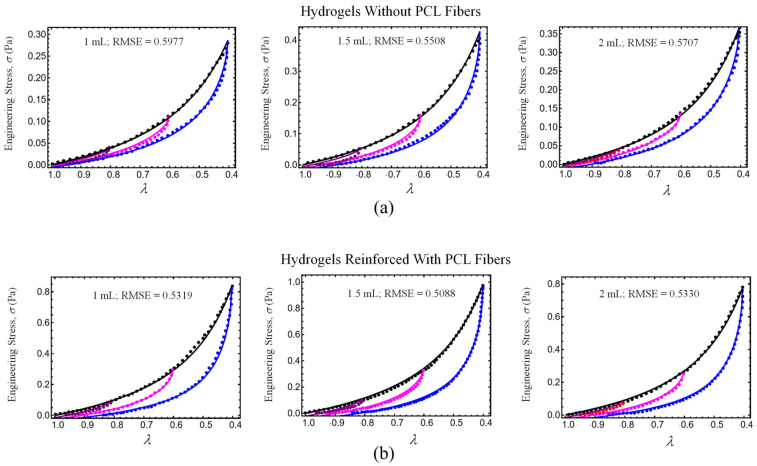
Loading and unloading engineering stress vs. stretch curves. (**a**) Hydrogels without fibers. (**b**) Hydrogels reinforced with fibers. Color lines represent theoretical predictions obtained from Equations (7) and (8). Dots are experimental data collected from compressive tests.

We shall consider experimental data collected from hydrogel specimens mixed with 1 mL, 1.5 mL, and 2 mL of gentamicin sulfate added as a crosslinker with and without reinforcement and compare these with predicted values obtained from Equations (7) and (8). As can be seen in Table 4, the shear modulus of the hydrogel specimens, $\mu_{0}$, and the chain number of links, $N_{8}$, are first determined from the virgin loading curved using Equation (7). With these values of the virgin material, we next use Equation (8) and the unloading stress-softened data to best fit the material constants $A_{1}$, $A_{2}$, b, and d. The results predicted from our material model given by expressions (7) and (8) are shown in Figure 15a. Notice that when the hydrogels are reinforced with fibers, their strength increases by more than 250%, as illustrated in Figure 15b. We use the root-mean-square error (RMSE) value to evaluate the accuracy of our theoretical prediction, which is obtained from Equations (7) and (8). The corresponding RMSE values are deployed in each curve plotted in Figure 9, Figure 10, Figure 11 and Figure 15.

We also use Equation (7) to compare theoretical engineering stress versus stretch curves to experimental data collected from the patellofemoral groove of an equine [65] and of a knee joint and human chondral cartilage knee layer [127] plotted in Figure 9. The corresponding parameter values used to plot the dashed color lines in Equation (7) were μ_0_ = 0.2 MPa, N_8_ = 10, f = 0.16, A_1_ = −1.35 MPa, A_2_ = 7, and μ_0_ = 0.2 MPa, N_8_ = 5, f = 0.125, A_1_ = −1.9 MPa, A_2_ = 14.5 MPa for the equine and human cartilage, respectively. Notice that the predicted results follow experimental data well.

## 5. Conclusions

This study demonstrated the successful development of reinforced PVA/GL/CH hydrogels for cartilage tissue engineering, using gentamicin sulfate as an antimicrobial agent and crosslinker and polycaprolactone (PCL) fiber for mechanical reinforcement.

In summary, the following main findings of our study are the following:

- Hydrogels crosslinked with GS exhibited improved tensile and compressive performance. The HT-AB samples achieved an ultimate tensile strength of 1.768 MPa and a compressive strength of 1.196 MPa, compared with 1.389 MPa and 0.921 MPa in non-GS (HT-C) samples. The LT-AB hydrogels showed a 49.2% increase in tensile strength and a 244.75% increase in compressive strength relative to their LT-C counterparts.
- FTIR spectroscopy revealed changes near 3000 cm^−1^, indicating hydrogen bonding between GS and polymer components. An additional absorption band around 3325 cm^−1^ further supported interactions with hydroxyl and amine groups. Thermal analysis showed enhanced stability in GS-crosslinked hydrogels, with maximum decomposition temperatures increasing to 380.2 °C and 445.3 °C in HT-AB samples, compared with 312.7 °C in HT-C samples.
- SEM imaging confirmed uniform PCL fiber distribution and integration into the hydrogel matrix. Surface roughness values of 2.54 µm (HT-AB) and 2.74 µm (LT-AB) closely matched the 1–6 µm range of native articular cartilage. Fiber inclusion did not compromise water retention, and LT-AB samples retained water more effectively over 29 h than other formulations.
- XRD measurements showed that the gelation method does not influence the crystallographic properties of HT- and LT-hydrogels.
- A non-Gaussian strain energy density model accurately reproduced the hydrogel’s mechanical response under uniaxial loading/unloading conditions. The predicted hydrogel shear modulus values closely matched the human cartilage values previously reported in the literature.
- The root-mean-square error (RMSE) value displayed in each curved plotted in Figure 9, Figure 10, Figure 11 and Figure 15 indicate that theoretical predictions follow experimental data well.

The findings reveal the promising potential of the engineered PVA/GL/CH hydrogels, enhanced with GS and PCL fibers, for cartilage implants and tissue engineering applications. These remarkable properties shed light on how to contribute to the advancement of developing novel hydrogels in joint repair and cartilage replacement.

## Figures and Tables

**Table 1:** TGA and DTGA parameters for hydrogels and their precursors.

Sample	T_10%_ (°C)	T_p1_ (°C)	T_p2_ (°C)	R_p1_ (%/°C)	R_p2_ (%/°C)	Residue (%)
Chitosan	286.3	78.3	310.5	0.10	1.03	39.8
Gelatin	275.3	94.2	340.5	0.08	0.60	30.0
PVA	280.5	290.0	321.7	1.01	0.91	5.6
HT-C	63.1	70.6	312.7	0.91	0.43	7.0
HT-AB	266.4	380.2	445.3	0.66	0.46	7.7
LT-C	60.1	66.2	314.5	1.12	0.54	6.9

**Table 2:** Average roughness R_a_ parameter of some reported works and experimental hydrogels.

Sample	Ra (μm)	Reference
Articular cartilage	1.0–6.0	[120]
PVA + Sodium Dodecyl Sulfate hydrogel	21.04 ± 0.1	[121]
HT-AB	2.5	This work
LT-AB	2.7	This work

**Table 3:** Mechanical properties for the hydrogel synthesized and some published references.

Material	Crosslinker	Ultimate Stress (MPa)	Compressive Stress (MPA)	Reference
HT-C	Dried at 50 °C	1.389	0.921	This work
HT-AB	GS, dried at 50 °C	1.768	1.196	This work
LT-C	Freeze/Thaw Cycles	0.203	0.038	This work
LT-AB	GS, Freeze/Thaw Cycles	0.303	0.131	This work
PVA	Freeze/Thaw Cycles	0.024 ± 0.012	---	[136]
PVA/Gelatin	Freeze/Thaw Cycles	0.92 ± 0.018	---	[64]
Chitosan/PVA	Glutaraldehyde	Fracture stress: 196.82	---	[121]
GelMa reinforced 93%	APS/TEMED		0.05	[65]
PVA/Chitosan	Irradiation		0.072	[137]
Human knee cartilage	---		0.051	[65,127]

**Table 4:** Material parameter values to fit the mathematical model with experimental data. G: Gentamicin, w: without reinforcement, r: reinforcement.

Specimen	*µ*_0_ (MPa)	*N* (-)	*A*_1_ (MPa)	*A*_2_ (MPa)	*b* (-)	*C* (MPa)	*f* (-)
G1w	0.23	20	0.45	−0.05	0.5	−120.25	0.04
G1.5w	0.25	25	−4.175	0	0.65	−40.25	0.06
G2w	0.25	20	−3.31	0	0.475	−60.25	0.0787
G1r	0.25	5.75	−4.75	0	0.85	−15.25	0.04
G1.5r	0.25	8.25	−2.2	0	0.85	−10.25	0.06
G2r	0.25	7.25	−2.25	0	0.85	−15.25	0.0787

## Data Availability

Data will be made available on request.
